# Real-world effectiveness and tolerability of small-cell lung cancer (SCLC) treatments: A systematic literature review (SLR)

**DOI:** 10.1371/journal.pone.0219622

**Published:** 2019-07-18

**Authors:** Manca Povsic, Ashley Enstone, Robin Wyn, Klaudia Kornalska, John R. Penrod, Yong Yuan

**Affiliations:** 1 Adelphi Values, Bollington, Cheshire, United Kingdom; 2 Bristol-Myers Squibb, Princeton, NJ, United States of America; University of Mississippi Medical Center, UNITED STATES

## Abstract

**Objectives:**

SCLC makes up approximately 15% of all lung carcinomas and is characterized by relatively aggressive spread and poorer prognosis compared to other lung cancers. Treatment options are limited, and their efficacy in randomized trials is poor, whilst outcomes in clinical practice remain unclear. The aim of this study was to assess the real-world effectiveness and tolerability of SCLC treatments.

**Methods:**

An SLR was conducted across nine databases accessed through OVID, capturing observational, non-randomized studies published between 01/2006–11/2018. In total, 554 abstracts were retrieved and systematically screened for eligibility. The eligible publications included effectiveness and tolerability data from adult SCLC patients (at any line of therapy). Additional grey literature searches were conducted.

**Results:**

Forty-three publications were included in this review—data from first-line therapies were captured most often (n = 32), while data from second (n = 14) and third line (n = 7) and beyond (n = 7) were less frequent. The publications reported primarily on chemotherapy/radiotherapy. The majority of publications lacked robustness and only 14/43 conducted statistical analyses or controlled for bias. Median OS for the largest SCLC populations were 9.6 months at first line (n = 23,535) and 4.9 months at second line (n = 254) for treatment with chemotherapy, and 4.7 months at third line (n = 120) for predominantly platinum-based chemotherapy or cyclophosphamide/adriamycin/vincristine. Hematologic toxicities (such as neutropenia, thrombocytopenia and anemia) were the most frequently reported TRAEs (n = 9).

**Conclusions:**

Real-world treatment effectiveness and tolerability data were fragmented and inconsistently reported, and available publications were primarily of poor quality and lacked statistical analyses. This SLR showed limited treatment options and poor OS in SCLC, with no treatment option being clearly superior. TRAEs additionally increased the burden of this already challenging disease. Recent data suggest real-world outcomes are even poorer that those reported in clinical trials, and that novel therapies are needed to offer new treatment options for patients.

## 1 Introduction

Small-cell lung cancer (SCLC) is an aggressive type of lung carcinoma,[1] comprising approximately 13% of all new lung cancer diagnoses[2, 3] and accounting for more than 180,000 cases worldwide per year.[4] It is characterized by late diagnosis, high frequency of recurrence and poor survival.[2, 3] SCLC presents either as limited disease (LD; where cancer is contained to one side of the chest and can be adequately encompassed in a radiation port), or extensive disease (ED; where cancer has spread further through the body and cannot be encompassed in a radiation port).[5] Generally, two out of three SCLC patients present with ED at diagnosis;[3] therefore, the majority of patients are diagnosed after the disease has already metastasized, limiting treatment options. There has been a modest but significant improvement in median survival time and 5-year survival rate over the last 30 years for LD-SCLC patients;[6] however, these outcomes remain poor in ED-SCLC patients.[4, 7]

Platinum- (cisplatin or carboplatin) and etoposide- or irinotecan-based regimens are the mainstay of SCLC first-line therapy.[4] However, discrepancies exist in the comparative efficacy of these treatments: recent meta-analyses of randomized controlled trial (RCT) data have shown that platinum chemotherapy in combination with irinotecan increases overall survival (OS), progression-free survival (PFS), objective response rate (ORR) and 1-year survival in ED-SCLC, compared to platinum/etoposide regimens.[8, 9] In contrast, a similar meta-analysis did not report any ORR or disease control rate improvements with the same regimen, providing little conclusive data on the clear superiority of one treatment over the others.[10] Despite these results, the survival benefit reported in RCTs remains modest regardless of the treatment type used.[11, 12]

Topotecan or amrubicin, and a combination of cyclophosphamide, doxorubicin and vincristine (CAV) constitute second-line therapy;[2] however, these chemotherapies have not conclusively demonstrated superiority over topotecan in a RCT setting, which itself provides only modest outcomes.[13, 14] In third line and beyond, there is a paucity of high-quality evidence to guide treatment decisions.[15] Additionally, the rates of relapse of SCLC within two years are high and the prognosis with current treatments remains poor across all lines of therapy, with the median survival time reported in RCTs varying from 12.9 to 20 months in first-line patients with LD-SCLC and from 7.9 to 12.8 months in first-line patients with ED-SCLC, both treated with platinum-based regimens.[11, 12] In second line, RCTs report a median survival time between 15 and 20 months for LD-SCLC, and 9.4 to 12.8 months for patients with ED-SCLC, both treated with platinum-based regimens.[2] Recent literature suggests that real-world outcomes in cancer patients are equally modest, with surrogate outcomes being even poorer than reported in RCTs.[16] It is expected that SCLC patient outcomes in the real world will be equally poor.

Few new SCLC treatments have become available over the past two decades. Notably, nivolumab monotherapy has been approved by the Food and Drug Administration (FDA) in August 2018 for third-line treatment of metastatic SCLC due to its durable response and good tolerability.[17, 18] Similarly, atezolizumab has been granted priority review by FDA in December 2018, and when combined with chemotherapy, it showed an improved OS and PFS in treatment-naïve patients with ED-SCLC.[19, 20] Although new therapies have shown improved outcomes in RCTs,[18, 20] their effectiveness and tolerability in the real world is unknown.[21]

There is a need for robust evidence on comparative treatment efficacy and safety in SCLC, specifically by line of therapy. To aid decision-makers in selecting the optimal treatment for each patient, the *effectiveness* of new and existing therapies (in the real world), as well as their *efficacy* (in RCTs) should be studied. However, availability of these data, particularly for novel therapies, is currently limited. This systematic literature review (SLR) was conducted to examine the real-world effectiveness and tolerability of SCLC therapies. The primary objective was to understand the relative effectiveness, tolerability and health-related quality of life (HRQoL) impact of interventions in SCLC, including those at first line and subsequent lines of therapy (e.g. second-line, third-line, maintenance, consolidation), as well as platinum-sensitive and refractory therapy, as shown by real-world evidence. The secondary objectives were to understand SCLC therapy effectiveness, tolerability and HRQoL impacts in any subpopulations of SCLC patients, including LD- and ED-SCLC patients, programmed death-ligand 1-positive and -negative patients, and patients with brain metastases associated with SCLC.

## 2 Methods

An SLR was performed to identify publications relating to the above objectives in the real-world setting, in line with Cochrane and York Centre for Reviews and Dissemination guidelines for conducting reviews,[22, 23] and Preferred Reporting Items for Systematic Reviews and Meta-Analyses (PRISMA) guidelines for reporting. Published literature was captured through searches of OVID-indexed databases: MEDLINE, Embase, Econlit, PsycINFO, and Evidence-Based Medicines Reviews databases (Cochrane Central Register of Controlled Trials, Cochrane Database of Systematic Reviews, Database of Abstracts of Reviews of Effects, Health Technology Assessment database, and the National Health Service Economic Evaluation Database). We have conducted a simultaneous search across all nine included databases, followed by application of limitations and deduplication. One search strategy was used across all databases—the search terms irrelevant to any specific databases were automatically removed via the OVID search engine.

In order to capture all relevant evidence, two searches were conducted investigating clinical and HRQoL endpoints in the real world. The two searches were originally conducted on the 12^th^ February 2018 and updated on the 19^th^ November 2018 to include all newly published studies (for search strategies see S1 and S2 Tables). These searches were limited to English-language publications and included literature published in the last 12 years (January 2006 to November 2018). Included publications reported on adult (≥18 years) SCLC patients who received immune-therapy, single-agent or combination chemotherapy, or radiotherapy, and presented outcomes of interest: OS, PFS, ORR (comprising complete response [CR]/partial response [PR], stable disease [SD]/progressive disease [PD]), death/mortality, time to response, time to progression, duration of response, treatment-related adverse events (TRAEs), TRAEs leading to discontinuation, or HRQoL. Publications from interventional studies were excluded.

To supplement the records captured through OVID, an additional grey literature search was conducted through a manual search of conference proceedings, including those from the American Association for Respiratory Care, the American Society of Clinical Oncology, the European Lung Cancer Congress, the European Society for Medical Oncology, the International Association for the Study of Lung Cancer, and the International Society for Pharmacoeconomics and Outcomes Research. The following keywords were used to conduct these searches: “SCLC”, “small-cell”, “small cell” and “oat”. The results were then manually searched for outcomes of interest. Further records were obtained through online trial registries (clinicaltrials.gov and the World Health Organization International Clinical Trials Registry Platform) to identify observational studies, and via Google and recursive searching (reference search of relevant publications), using the same keywords as previously applied for the grey literature search.

All identified abstracts were screened for full-text inclusion by two independent reviewers (KK and RW). Any disagreement between the reviewers was discussed and the reasoning for specific disagreements was stated. This reasoning was then reviewed by a senior researcher (MP), who made the final decision on inclusion or exclusion of the record.

In order to ensure good internal agreement when screening, the Cohen’s Kappa was calculated. This statistical method is a measure of the extent to which the reviewers assigned the same score to the same variable, where a measure of 1.0 signifies complete agreement and 0.0 signifies no agreement.[24] The Cohen’s Kappa for abstract screening and data extraction were reported as 0.85 and 1.0, respectively, suggesting a high agreement between the reviewers.

A quality assessment of the included peer-reviewed publications was conducted using the Risk of Bias in Non-Randomized Studies of Interventions questionnaire (ROBINS-I).[25] The questionnaire was based on assessing the quality of broad themes of bias, categorized into domains. Depending on the risk of bias within each domain, a final ‘score’ (mild, moderate, serious) was estimated for each publication. The robustness of the identified publications was also assessed through examination of the statistical analyses (or their absence) used to control for bias. Publications that were deemed of insufficient quality (population size <20 patients, unclear description of methodology) were excluded. Each included publication was stratified by the presented line(s) of therapy, with this characteristic being inferred (using intervention or demographic data) if not clearly stated in the text. Relevant data were extracted independently by two reviewers, and confirmed by a senior researcher.

## 3 Results

### 3.1 Search results

The clinical and HRQoL searches of the OVID-indexed literature captured 554 unique records for the time period between January 2006 and November 2018. Following screening by title and abstract, 525 records were excluded, primarily based on the exclusion criteria of incorrect population or study design. The remaining 29 records were subjected to full-text screening, and 10 records were excluded because of: irrelevant outcomes (i.e. not defined in search protocol); interventional study design; a small SCLC population size (n<20); or presenting duplicated results. Nineteen publications from OVID databases were thus included in this review.

Seven additional full text publications were sourced through recursive searching of the reference lists of publications captured from OVID. Seventeen grey literature records were also included: 10 records were retrieved from conference proceedings, two from online trial registries, and five through further internet-based searching.

**Fig 1:**
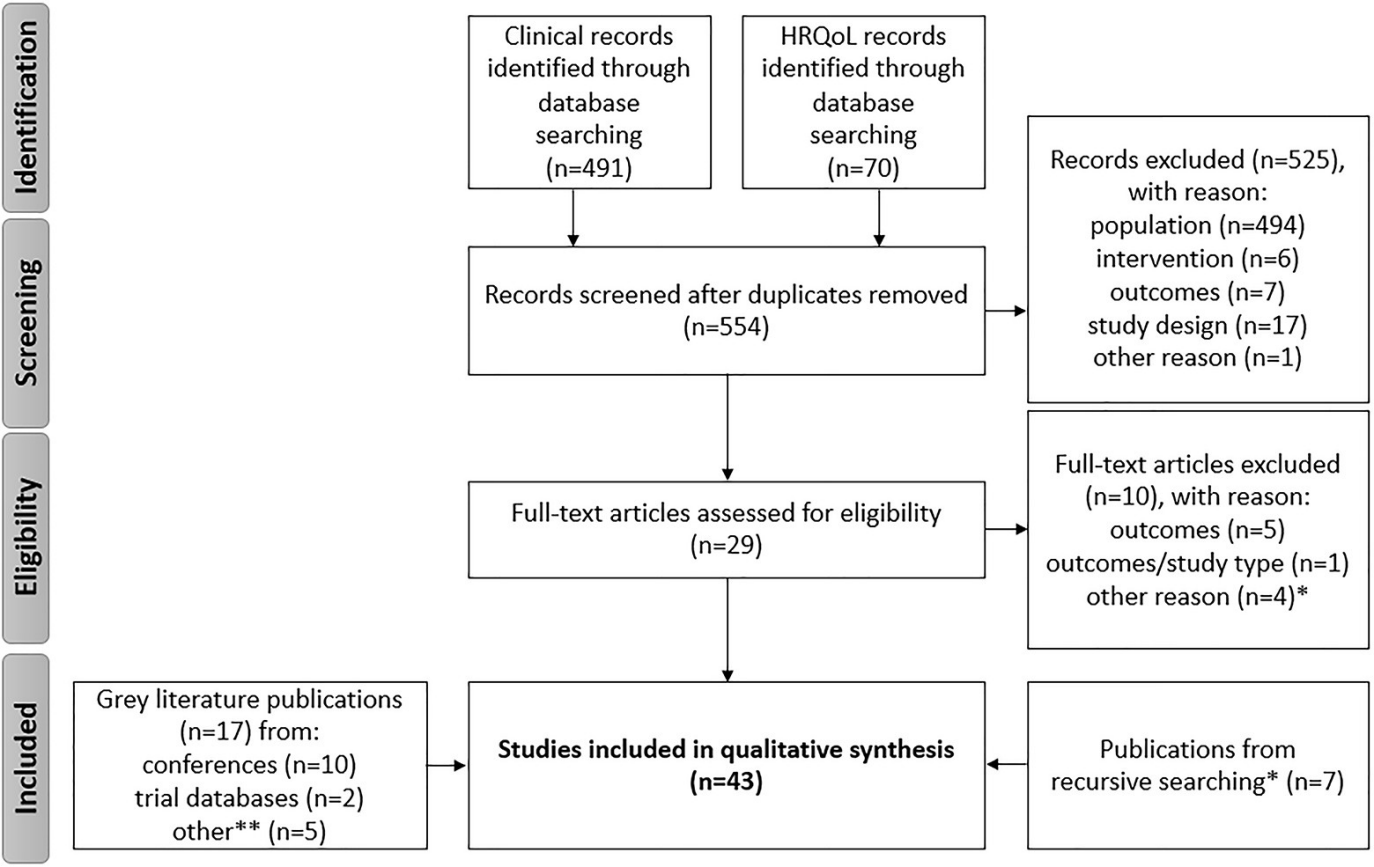
PRISMA flow diagram of captured publications. *Other reasons for exclusion at full-text screening stage were small population size (<20 SCLC patients) or duplicating results that had already been captured. **“Other” grey literature items were sourced through internet searches. HRQoL = health-related quality of life; n = number of records; PRISMA = Preferred Reporting Items for Systematic Reviews and Meta-Analyses.

In total, 43 publications were included in this review. Fig 1 presents the full SLR process flow, in accordance with PRISMA guidelines.[26]

### 3.2 Overview of outcomes

The searches yielded a total of 20 peer-reviewed full text publications and 23 abstracts or poster publications.[15, 27–68] Median OS and TRAEs were the most frequently and consistently reported outcomes in the real world, and thus, these data were the most comparable among the captured treatments, relative to other outcomes. While other data were reported frequently, the methods of measuring differed greatly, including the timepoints of measure, the populations being measured, and the definitions used.

Table 1 summarizes the number of publications reporting on each outcome across all SCLC populations. Although HRQoL outcomes were a target of this review, no studies reporting relevant HRQoL data were captured.

**Table 1:** Number of publications reporting each outcome in the SCLC population, per line of therapy.

**OS**	**PFS**	**ORR**	**Mortality/landmark survival**	**TTP**	**Duration of response**	**TTR**	**TRAEs**	**TRAEs leading to discontinuation**	**HRQoL**
**First-line therapy (n = 32)[15, 27–35, 37–42, 44, 50–52, 54–56, 58, 59, 61–67]**									
21	7	15	14	2	1	0	8	3	NR
**Second-line therapy (n = 14)[15, 33, 36, 40, 43, 45, 46, 52–54, 59, 62, 63, 68]**									
9	5	7	4	1	NR	NR	3	2	NR
**Third-line therapy (n = 7)[15, 49, 52–54, 60, 63]**									
6	2	3	4	NR	NR	NR	1	1	NR
**Mixed lines (n = 7)[47, 48, 54, 57, 63, 65, 68]**									
7	4	3	2	NR	1	NR	2	1	NR

HRQoL = health-related quality of life; n = number of records; NR = not reported; ORR = objective response rate; OS = overall survival; PFS = progression-free survival; SCLC = small-cell lung cancer; TRAEs = treatment-related adverse events; TTP = time to progression; TTR = time to response.

Owing to the large quantity of outcomes data found, only median OS and TRAE data for overall populations (containing both LD- and ED-SCLC cases) are reported in this manuscript, from 22 publications in total, including 11 full-text publications and 11 conference abstracts or posters.[15, 28–30, 34–38, 42–44, 47, 49, 51, 52, 54, 57, 60, 62, 63, 68] These outcomes and population were selected based on the following rationale:

- OS is a direct measure of clinical benefit of the selected treatments and is the standard efficacy outcome, as opposed to surrogate outcomes (e.g. PFS or ORR).[69, 70]
- A key objective of this SLR was to investigate safety, and TRAE outcomes provide information about the safety, tolerability, and HRQoL impact of the administered treatments.
- The majority of studies reported on the overall population (i.e. a mixture of both LD and ED), and due to the nature of the real-world data reported, the results were not frequently stratified by stage. Where results were stratified, the data were fragmented and less comparable.

The full details of each included publication are presented in S3 Table. Included publications were grouped according to the presented line of therapy for the purpose of outcomes comparison and analysis. Where possible, information about line of therapy was extracted directly from the text of the publication.[15, 30, 34–37, 39, 49, 51, 52, 54, 57, 60, 62, 63, 68] However, for publications where this was not specified, this was inferred based on available information (S4 Table).[27–29, 38, 42–44, 47]

### 3.3 Quality assessment and statistical analysis

Eleven peer-reviewed publications were assessed for quality through the ROBINS-I tool. Other publications (e.g. conference abstracts and posters) provided only limited information on the methodologies used therefore, the quality of these publications could not be measured.

Three full-text publications were judged to have a mild risk of bias,[15, 36, 63] while six had moderate risk,[28, 34, 35, 37, 52, 62] and two had serious risk.[29, 30] The moderate or serious risk of bias was most often due to the missing data, confounding factors, deviations from the intended interventions and unclear definitions of outcome measurements. Additionally, there was a lack of complete reporting of the results across all publications, with only three publications explicitly stating the duration of follow-up.[30, 34, 35]

All full-text publications presenting with the moderate or serious risk of bias (n = 8) lacked information about how the researchers controlled for missing data.[28–30, 34, 35, 37, 52, 62] In the captured real-world studies, treatment selection was always based on the opinion of a treating physician and, therefore, could not be controlled for.

In addition to bias assessment via ROBINS-I, we also conducted an analysis of the statistical methods used in the captured publications. Fourteen of the included publications reported a form of statistical analysis.[15, 28–30, 34–37, 51, 52, 57, 60, 62, 63] Statistical analysis used to establish the comparability of patient demographics was conducted in four publications, and included Wilcoxon rank-sum test, Fisher’s exact test, Mann-Whitney U-test and χ^2^ test.[28, 29, 34, 52] Sixteen publications did not conduct any type of statistical analysis on patient demographics,[15, 30, 36–38, 42–44, 47, 49, 54, 57, 60, 62, 63, 68] while two publications did not specify what type of analysis was conducted.[35, 51] However, most publications (n = 13) included statistical analysis of outcomes data.[15, 28–30, 34–37, 51, 57, 60, 62, 63] Within these, the most commonly used statistical methods included logistic regression modelling, Cox proportional hazard modelling, and Kaplan-Meier analysis with log-rank testing. Conversely, nine publications did not conduct any statistical analysis, or did not outline the type of analysis that was conducted.[38, 42–44, 47, 49, 52, 54, 68] Full details of analyses conducted in each publication are presented in S3 Table.

### 3.4 First-line therapy

First line outcomes were the most frequently captured in the real world: ten publications reported on median OS and/or TRAEs.[28–30, 34, 35, 37, 38, 42, 44, 51] In all publications, chemotherapy was the main SCLC treatment administered,[28, 30, 34, 35, 37, 38, 44, 51] and in all but two publications, platinum-based regimens were the most common treatments of choice.[30, 34, 35, 38, 44, 51] Despite the fragmented nature of the available data, the following trends were identified. Median OS in chemotherapy-treated first-line patients rarely exceeded one year.

#### 3.4.1 Median OS in chemotherapy-treated first-line patients rarely exceeded one year

**Fig 2:**
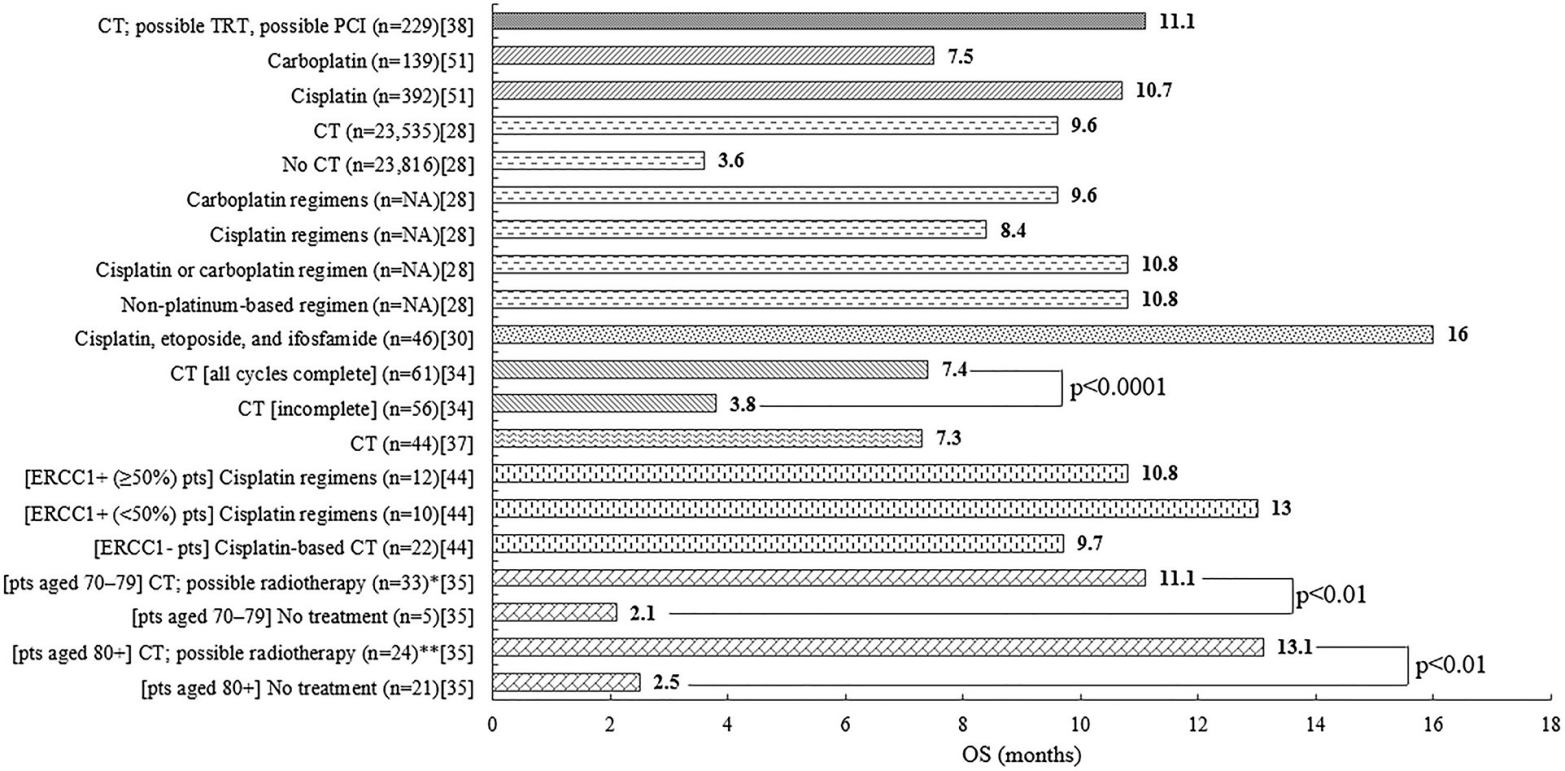
Median OS (months) at first line[28, 30, 34, 35, 37, 38, 44, 51]. *One patient received radiotherapy only. **Three patients received radiotherapy only. CT = chemotherapy; ERCC1- = excision repair cross-complementation group 1 negative; ERCC1+ = excision repair cross-complementation group 1 gene positive; n = number of patients; NA = not available; OS = overall survival; PCI = prophylactic cranial irradiation; pts = patients; TRT = thoracic radiotherapy.

Median OS for patients treated with chemotherapy in the real world ranged from 7.3 to 16 months (Fig 2).[28, 30, 34, 35, 37, 38, 44, 51] Median OS did not appear to be substantially affected by patients’ age group,[35] or by presence of the excision repair cross-complementation group 1 (ERCC1) biomarker,[44] which has previously been used to help select therapy in non-small-cell lung cancer. The highest OS was seen among consecutive patients receiving cisplatin, etoposide, and ifosfamide treatment at a single center (16 months, Fig 2).[30] However, the study patients (n = 46) were relatively young and had a good performance status,[30] and the process of measuring OS in this study (from diagnosis rather than treatment initiation) may also have contributed to the apparent effectiveness of this regimen.[30] The lowest OS for complete treatment was reported in patients receiving inpatient urgent chemotherapy for advanced solid tumors on the medical oncology unit.[37] This group of patients (n = 44) survived for 7.3 months; however, this relatively low OS may have been caused by the advanced nature of disease.[37]

There was little agreement regarding whether specific chemotherapy regimens led to significantly different median OS in the real world.[28, 51] A large-scale registry study including 23,535 patients treated with chemotherapy did not report significantly different median OS between patients treated with carboplatin or cisplatin regimens (p = 0.775), or between patients treated with platinum or non-platinum regimens (p = 0.237).[28] In contrast, a registry study including 531 patients treated with carboplatin or cisplatin regimens reported substantially different median OS for these therapies: 7.5 months versus 10.7 months, respectively; however, statistical analysis was not provided.[51]

Although the comparison of the benefits of different regimens was inconclusive, patients who received chemotherapy had longer median OS than those who did not (p<0.001),[28] and complete chemotherapy led to longer median OS than incomplete chemotherapy (p<0.0001).[34] While an analysis of associations between demographic groups and treatments, as well as statistical analysis of the results was conducted, the circular relationship between OS and the completion of chemotherapy was not examined.[34] However, the authors did conclude that elderly patients are able to withstand the chemotherapy and receive a survival benefit from the treatment.[34]

Additionally, radiotherapy was specifically described,[28, 35, 38] or otherwise allowed or expected [30, 34, 44] in all but one study; however, no median OS data were available to determine the effect of adding radiotherapy to chemotherapy at first line.

#### 3.4.2 Hematological toxicities were the most frequently reported TRAEs at first line

**Fig 3:**
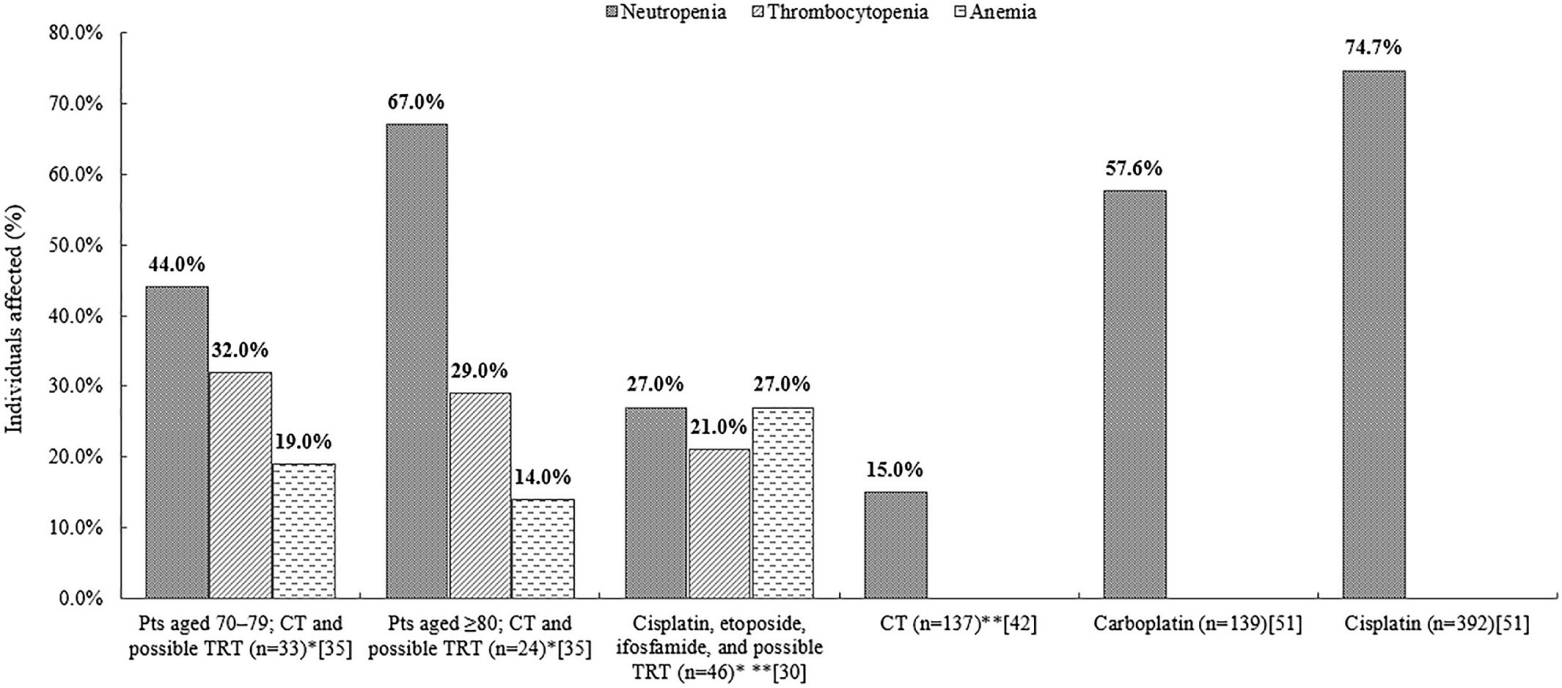
Most common TRAEs reported for patients treated with SCLC therapies[30, 35, 42, 51]. Note: Presented TRAEs exceed 5% or lead to discontinuation. *These TRAEs were specified as grade 3–4. **These outcomes refer to febrile neutropenia specifically. CT = chemotherapy; n = number of patients; pts = patients; SCLC = small-cell lung cancer; TRAEs = treatment-related adverse events; TRT = thoracic radiotherapy.

Six publications reported on TRAEs following first-line therapy.[29, 30, 34, 35, 42, 51] All but one publication reported hematological toxicities, e.g. neutropenia, leukopenia, thrombocytopenia or anemia.[30, 34, 35, 42, 51] The remaining publication recorded patient-reported adverse events and the experienced symptoms such as fatigue, skin rash or hair loss.[29] Fig 3 presents the frequency of hematological toxicities in the captured publications at first line.

The majority of studies (n = 3) at first line did not specify the severity of reported TRAEs.[34, 42, 51] The two captured studies that did specify this reported the severity as grade 3–4.[30, 35]

Captured studies reported neutropenia as the most common TRAE.[30, 35, 42, 51] The highest incidence was reported in a retrospective chart review of SCLC patients (n = 531) receiving cisplatin or carboplatin, where patients receiving cisplatin had a significantly higher incidence of neutropenia versus patients receiving carboplatin (74.7% versus 57.6%; p<0.01).[51] High incidence was also reported in another retrospective chart review, where very elderly SCLC patients (aged ≥80) who received chemotherapy with possible thoracic radiotherapy (TRT, n = 45) experienced a higher incidence of neutropenia, versus a younger (aged ≥70; n = 38) population (67% versus 44%; no p-value available as statistical analysis was not conducted for this comparison).[35] Only two out of the captured studies reported on febrile neutropenia specifically, noting the incidence at 27% in patients receiving cisplatin, etoposide, and ifosfamide with possible radiotherapy; and 15% in patients treated predominantly with platinum and etoposide therapy.[30, 42] No other studies reported on febrile neutropenia and did not specifically report on cases of fever in patients with neutropenia.[35, 51]

Thrombocytopenia and anemia were also commonly reported across first line.[30, 35] The incidence of thrombocytopenia found in SCLC patients aged ≥70 years and ≥80 years receiving chemotherapy and possible TRT was 32% and 29%, respectively (no p-value available as statistical analysis was not conducted for this comparison).[35] The incidence of anemia in those patient groups was 19% and 14%, respectively (no p-value available as statistical analysis was not conducted for this comparison).[35] Incidence of anemia reported in patients receiving cisplatin, etoposide, and ifosfamide with possible radiotherapy was 27%.[30]

Interestingly, no significant differences were observed in experienced TRAEs between elderly and very elderly patients, and researchers concluded that this may be caused by the observed frequent dose reductions or omissions, perhaps owing to a reluctance of both the physician and the older patient to risk severe toxic effects.[35]

Additionally, a retrospective registry study noted that hematological toxicities were the most common reason for dose reduction or treatment discontinuation.[34] Thirty out of 40 patients aged ≥75 years receiving chemotherapy had a dose reduction because of hematological toxicities, whilst 32 out of 56 patients discontinued their treatment owing to the same reasons.[34] Similarly, neutropenia was also a commonly reported reason for dose delays in patients with tumors.[42] Moreover, three toxicity-related deaths were reported in a retrospective chart review; two of these were caused by sepsis and febrile neutropenia, and one by a heart failure.[30]

The use of platelet transfusion or growth factors to manage treatment-associated toxicities was rarely reported across studies (n = 3).[30, 35, 42] Platelet transfusion was reported in cases of severe anemia and thrombocytopenia with chemotherapy and possible radiotherapy in patients aged ≥70 years; however, it was used rarely.[35] In cases of febrile neutropenia or thrombocytopenia requiring platelet transfusion, the administered treatments were reduced by 50% in patients treated with cisplatin, etoposide and ifosfamide with possible TRT, to minimize the toxicity associated with treatment.[30] However, research concluded that the dose reduction may have also reduced the relative effectiveness of the administered treatment.[71]

The administration of growth factors as a support intervention such as granulocyte colony-stimulating factors (G-CSF) was allowed in two studies; however, it was not included in the standard treatment regimen.[30, 35] When the use of G-CSFs was recorded as a primary prophylaxis for neutropenia management, it was reported that 32% of patients treated with chemotherapy (n = 137) received some type of G-CSF, and the reactive G-CSFs were the most commonly used type (27%).[42]

### 3.5 Second-line therapy

Substantially less data on SCLC outcomes were captured at second line than at first line.[36, 43, 62, 68] Three of the captured publications focused on chemotherapy outcomes,[36, 62, 68] and one focused on the effect of radiotherapy.[43]

#### 3.5.1 Second-line data were limited, and the treatment effects on median OS were inconclusive

**Fig 4:**
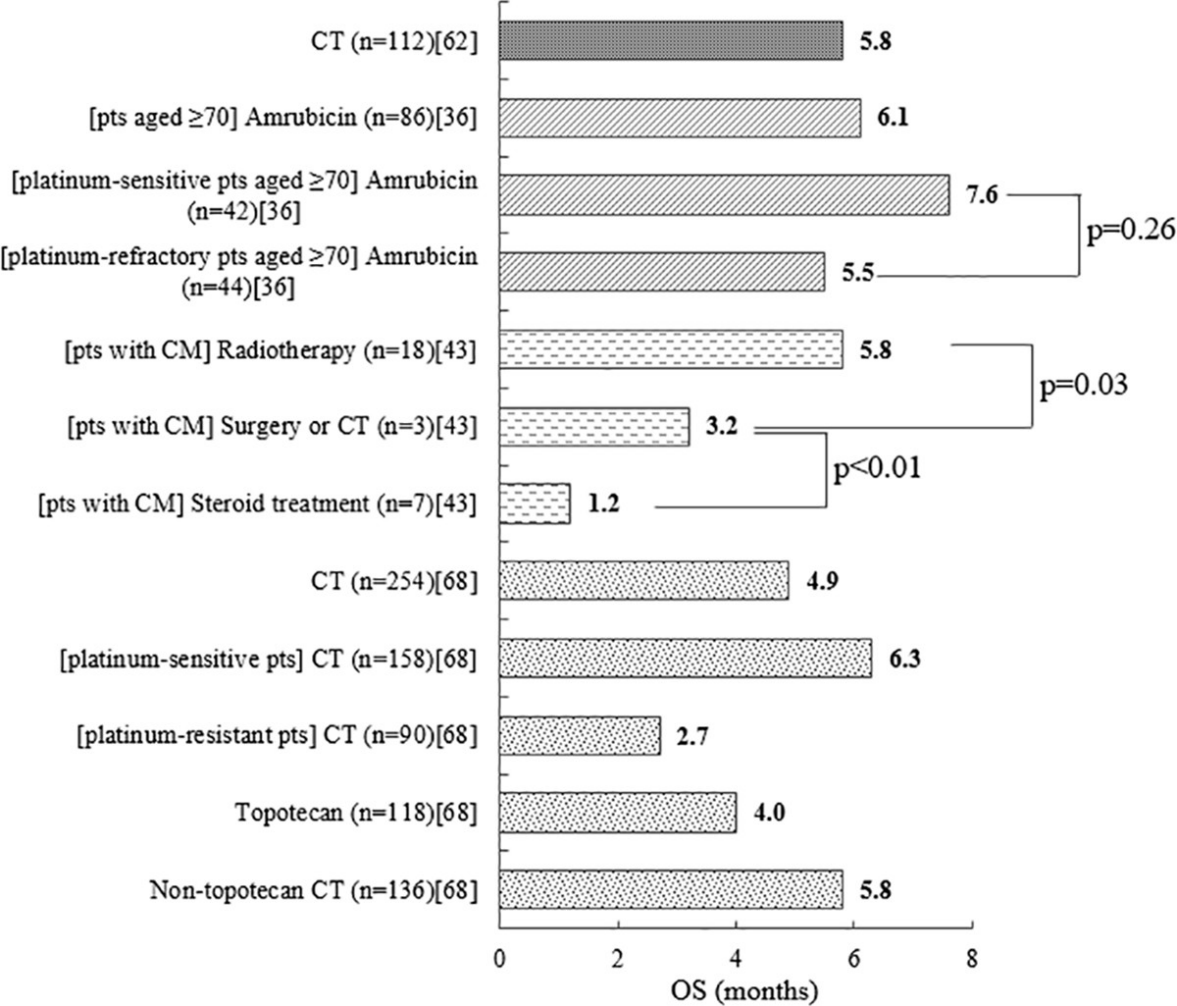
Median OS (months) at second line[36, 43, 62, 68]. CM = cranial metastasis; CT = chemotherapy; n = number of patients; OS = overall survival; pts = patients.

Median OS at second line ranged from 1.2 months in patients with cranial metastases treated with steroids to 7.6 months among platinum-sensitive patients (aged ≥70 years) treated with amrubicin (Fig 4).[36, 43, 62, 68]

No therapy was clearly associated with improved median OS at second line. A chart review of patients receiving varied second-line chemotherapies (n = 161) reported a median OS of 5.8 months; patients treated with platinum-based regimens had significantly superior OS relative to those treated with other chemotherapies (p = 0.004), but full results were not provided for context (Fig 4).[62] A registry study providing outcomes for patients treated with chemotherapy (n = 254) showed that patients who received topotecan had a lower median OS than those treated with non-topotecan therapies; however, statistical analysis was not provided (Fig 4).[68] This study compared the recent CheckMate 032 RCT outcomes data with a matched cohort of real-world patients selected through Flatiron database based on the CheckMate 032 eligibility criteria.[68] The result showed that patients treated with traditional chemotherapies in the real world setting had generally poor OS (4.9 months).[68]

The reported survival data for platinum-sensitive and platinum-refractory patients were also conflicting. In the above registry study, platinum-sensitive patients treated with chemotherapy showed better median OS outcomes than platinum-resistant patients, but statistical analysis was not provided (Fig 4).[68] In a further retrospective cohort study on patients treated with amrubicin at second line (n = 86), median OS was not significantly different between platinum-sensitive and platinum-refractory patients (p = 0.26).[36]

Only one study provided data for the effect of radiotherapy on OS.[43] In this chart review of prophylactic cranial irradiation (PCI)-treated patients (n = 28) developing cranial metastases at second line, re-irradiation led to significantly higher median OS relative to surgery/chemotherapy (p = 0.03) or steroid treatment (p<0.01) (Fig 4).[43]

#### 3.5.2 There was a paucity of literature reporting on TRAEs at second line

TRAEs were reported in only two retrospective chart reviews at second line.[36, 43] Hematological toxicities were the only TRAEs captured. Only one out of two captured studies reported the severity of TRAEs and this was specified as grade 3–4.[36] The most frequent TRAE was neutropenia, reported in 74.4% of patients (64 out of 86 evaluated) aged ≥70 years.[36] However, no studies reported on febrile neutropenia specifically. The incidence of thrombocytopenia and anemia was low in these patients (16.2% and 11.6%, respectively) and the authors concluded that amrubicin is a tolerable treatment for this population despite the high incidence of neutropenia.[36] The authors also reported that growth factors (G-CSF) were administered as a prophylactic agent against leukopenia and neutropenia at the physician’s discretion.[36] However, the exact information about its use in the management of toxicities was not reported.

Another retrospective chart review reported data on patients with brain metastases receiving PCI (n = 28); no prolonged neurologic toxicities were reported in this population.[43] Thus, PCI was concluded to be a safe treatment option in patients with brain metastases and limited life expectancy.[43]

### 3.6 Third-line therapy

Five publications reported median OS and/or TRAEs at third line in the real-world setting;[15, 49, 52, 60, 63] however, the data were fragmented, and only limited comparisons could be made. Each of these publications reported on outcomes of chemotherapy.[15, 49, 52, 60, 63]

#### 3.6.1 Median OS at third line was not significantly affected by choice of chemotherapy

**Fig 5:**
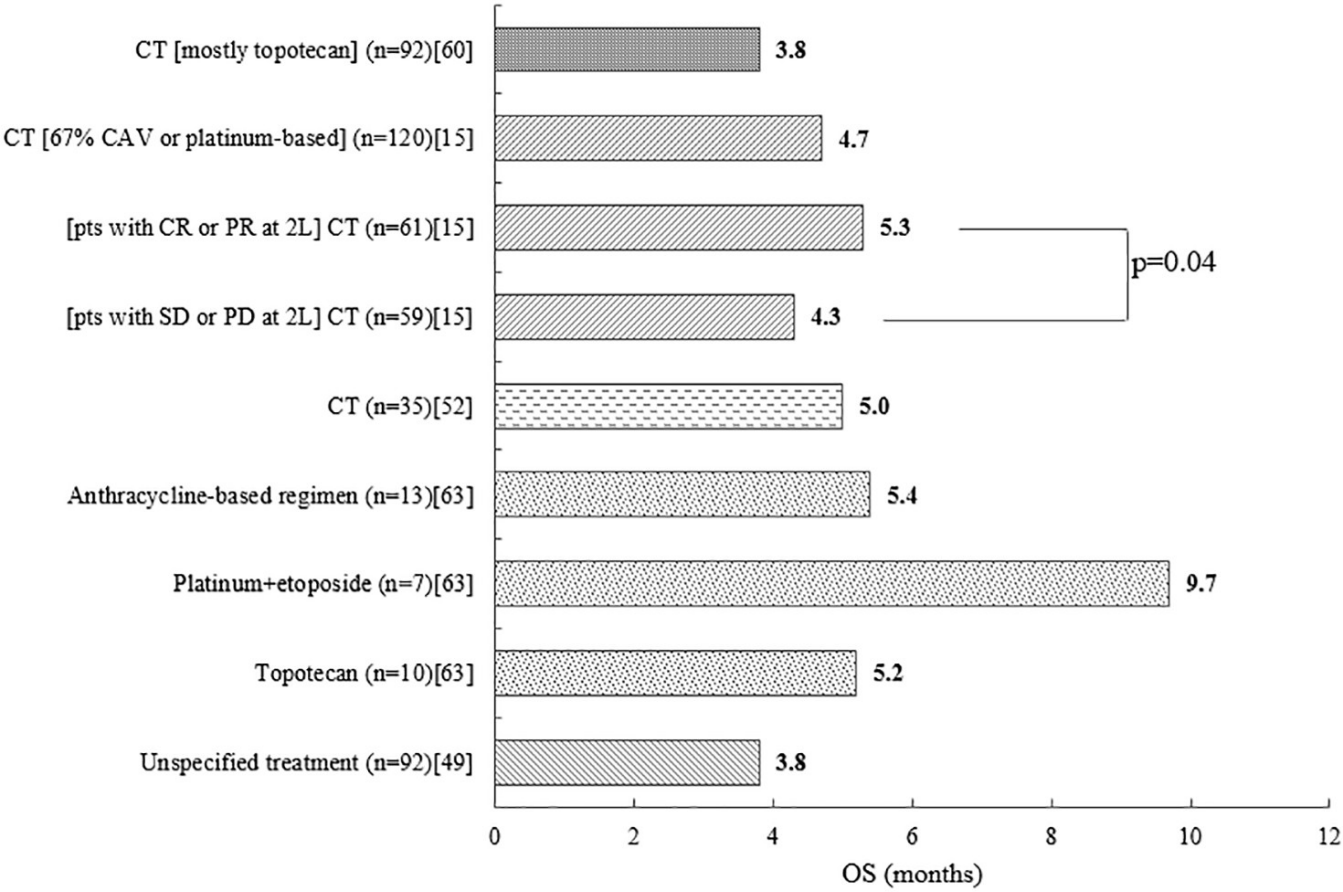
Median OS (months) at third line[15, 49, 52, 60, 63]. 2L = second line; CAV = cyclophosphamide, doxorubicin and vincristine; CR = complete response; CT = chemotherapy; n = number of patients; OS = overall survival; PD = progressive disease; PR = partial response; pts = patients; SD = stable disease.

Five studies reported outcomes for patients treated at third line, and the median OS ranged between 3.8 and 9.7 months (Fig 5).[15, 49, 52, 60, 63]

In a chart review (n = 25) which presented outcomes for different chemotherapy regimens, median OS was at least 5.2 months regardless of regimen (Fig 5).[63] There was no significant difference in median OS between patients who received anthracycline-based regimens, platinum and etoposide, or topotecan (p = 0.454).[63] Only one study reported a difference in the OS: patients (n = 120) who had CR or PR at second line had significantly higher median OS relative to those who had only SD or PD at that previous line (p = 0.04; Fig 5).[15]

No studies reported on the use of platelet transfusion or growth factors in relation to toxicities at third line.

### 3.7 Mixed lines of therapy

Five publications reported median OS and/or TRAEs data in patients at mixed lines of therapy (that is, data that could not be associated with individual lines).[47, 54, 57, 63, 68] The exact therapy administered was largely unspecified, but specific data were provided for platinum-based regimens, and paclitaxel.[47, 57, 63]

#### 3.7.1 OS values for mixed lines of therapy were fragmented and incomparable

The severely limited comparison of mixed-line outcomes was a consequence of the wide range of data captured.[47, 54, 57, 63, 68] Median OS was higher in publications containing first-line data,[54, 68] and lower in those containing data from later lines.[47, 57, 63] No studies provided mixed-line data comparing treatment regimens.

A registry study of patients who received first-line (and possible second- and third-line) therapy (n = 499), reported a median OS of 12 months, and stated that median OS did not differ substantially based on the time period in which each patient was treated.[54]

Outcomes data for paclitaxel were reported in two studies.[47, 57] A chart review of patients with interstitial lung disease (n = 21) receiving carboplatin and weekly paclitaxel, at third line or beyond, reported median OS of 7.1 months.[47] Conversely, a larger chart review of patients (n = 185) treated with paclitaxel alone, mainly at third or fourth lines, reported median OS of 3.3 months.[57]

#### 3.7.2 Paclitaxel-based regimens appeared to be well-tolerated throughout mixed lines

The above chart reviews reported on TRAEs for patients receiving paclitaxel as a part of their chemotherapy regimen.[47, 57] The first chart review of SCLC patients with interstitial lung disease (n = 21) receiving carboplatin and weekly paclitaxel reported an exacerbation of interstitial lung disease in 19% of individuals.[47] Two of the affected patients experienced a grade 5 interstitial lung disease; however the remaining patients experienced a ‘normal pattern’ of the disease.[47] The second chart review, of SCLC patients receiving paclitaxel alone (n = 185) reported fatigue (25%) and peripheral neuropathy (17%) as the most common TRAEs; however, 57% of dose reductions were caused by hematoxicities.[57]

No studies reported on the use of platelet transfusion or growth factors relating to toxicities across mixed lines of therapy.

## 4 Discussion

The primary finding of this SLR was the fragmentation of the effectiveness and tolerability data for SCLC patients in the real-world setting. Owing to the observational nature of the publications captured, a fundamental difference in study design, patient population, analysis timepoints, and lack of statistical analysis prevented any comparisons across publications. Where comparisons could be made within publications, use of different regimens did not consistently lead to significantly improved outcomes.

Eight out of the 11 quality assessed publications presented with a moderate or serious risk of bias, primarily due to missing data and a lack of information on how the authors controlled for these data.[28–30, 34, 35, 37, 52, 62] As all included studies were observational, which are inherently less robust than RCT studies, it was unsurprising that many of the captured publications exhibited a medium or high risk of bias. Although these publications exhibited a risk of selection bias, statistical comparisons of demographic or clinical characteristics across treatment subgroups were not always conducted, and the baseline comparability of subgroups could not always be established. However, the majority of included studies (n = 13) conducted statistical analyses of outcomes data; thereby increasing the robustness of conclusions. However, several publications included no adjusted analyses, and even when applied, this type of analysis may not fully avoid the risk of biases inherent to ‘real-world’ studies, e.g. physician choice, missing data, etc.

A majority of the captured outcomes data was related to first-line therapy, and a comparable depth of real-world outcomes data was lacking at second line and beyond. Median OS or TRAEs specifically were captured more frequently for first-line therapy publications (n = 10),[28–30, 34, 35, 37, 38, 42, 44, 51] than for second line (n = 4),[36, 43, 62, 68] third line (n = 5),[15, 49, 52, 60, 63] and mixed lines of therapy (n = 5).[47, 54, 57, 63, 68]

Despite the limited data comparisons available, some important trends were identified across publications: treatment options were limited and relied largely on well-established chemotherapy and radiotherapy options, with platinum-based regimens most commonly reported.[15, 28, 30, 44, 47, 51, 63] Relatively few patients were reported to receive third-line therapy and were often re-challenged with the types of treatment received previously.[15] In addition, several publications reported that a response to previous line of therapy can predict the response to subsequent lines.[62, 63, 68]

In this real-world setting, improvements provided by established treatments were limited, and the differences in median OS conferred by the various treatments were not always meaningful.[28, 63] At first line, OS rarely exceeded 12 months, and OS of patients in second line and beyond was rarely longer than six months.[36, 62, 68] Similar trends were recently observed in an SLR evaluating SCLC treatments in RCTs.[72] Evaluated chemotherapies showed poor outcomes overall, and no regimen was clearly superior to the others, suggesting little clinical difference exists across chemotherapy treatments in SCLC.[72] This shows the important unmet need in SCLC, particularly in already-treated patients.

Although both real-world and RCT outcomes appear poor, recent literature suggests that outcomes data in real-world clinical settings are worse than those from clinical trials. A matched comparison analysis of Surveillance, Epidemiology and End Results-Medicare database patients and patients included in RCTs (with similar baseline characteristics), who received similar treatment regimens, showed that the OS benefit of treatment in the real world was 16% lower than that predicted from RCT data.[16]

A publication included in this SLR contextualized clinical trial data through a comparison between RCT patients and a matched cohort of real-world patients created based on the trial inclusion criteria.[68] The results showed poor outcomes in real world patients and showed that patients treated with traditional chemotherapies in a real-world setting experienced generally worse survival than patients treated with immuno-therapies.[68] These data highlight the need for novel therapies that will provide a survival benefit in SCLC patients.

Real-world data are inherently inferior to the data produced in clinical trials; therefore, it is unsurprising that real-world results found in this SLR appear different to the previously reported RCTs. Patient outcomes in the real world may be worse than those seen in clinical trials due to patient populations included in clinical trials that are not reflective of the general population or clinical practice.[16] In addition, the provision of care and monitoring within a clinical trial does not always reflect the care that is seen in real-world clinics. Increased monitoring can be crucial in an aggressive disease such as SCLC, and thus may provide better outcomes in trials than in the real world.[16] Due to the inherent biases that exist in real-world studies, it is not possible to ascertain with certainty whether these real-world outcomes are genuinely worse than those in RCTs, or if this is an artefact of the study design, or a combination of both. Better designed real-world studies, with matched RCT cohorts and bias minimization are needed to examine this further.

Additionally, this SLR captured two patient registry studies reporting conflicting results.[28, 51] The discrepancies in the reported results could be due to significant differences in the examined populations, clinical practice and/or methodology.[28, 51] This showcases the inherent difficulties in comparing real-world data, as significantly different median OS in first line treatment was reported by a US population-based registry of 23,535 patients, versus a registry of 531 patients in the Manitoba province in Canada.[28, 51] Although these discrepancies may have been caused by the differences in the characteristics of target populations and variance in national standards of clinical practice, this is difficult to ascertain as one of the publications is an abstract, with limited information regarding patient population and treatment regimens.[51] A larger sample size generally suggests an improved validity, as larger registries provide an increased power for statistical analysis and detection of effect size. However, retrospective studies, such as registries, generally suffer from common biases such as selection and information biases, confounding factors or bias due to loss to follow-up, therefore the validity of the results of both registries could be diminished.[73]

To alleviate these biases and the variability caused by sample size, we have analyzed the statistical methods employed and the domains of highest bias across the captured publications and actively excluded studies from our analysis if they presented a low sample size (≤20 patients). Despite these measures, there were significant inconsistencies in the level of detail in the included study methodologies and the data from registries were generally of poor quality. This may have been reflected in the variances seen in the reported results and the reduced comparability between publications. Therefore, there is a clear need for a formalized methodology for registry studies, which would outline the minimum requirements for sample size, follow-up time and statistical analysis to be conducted in cancer registries, which would allow for increased comparability across real world data.

With regard to tolerability, no comparisons could be made between treatments. Hematological toxicities (neutropenia, thrombocytopenia, or anemia) were reported often, and at high rates, among patients receiving chemotherapy.[30, 35, 36, 42, 51, 52] Neutropenia was the most common TRAE, reported in ≤67% patients at first line[35] and up to 74% at second line, suggesting an increase in adverse event burden as patients move through therapy lines.[36] Paclitaxel-based regimens were often captured in mixed-line studies and appeared to be well tolerated; however, an exacerbation of interstitial lung disease is a possible risk in this already high-burden population.[47, 57] The use of platelet transfusion and growth factors relating to toxicities was rarely reported across publications, as only 4 out of 22 captured publications reported these data at first or second line.[30, 35, 36, 42] No publications reported on this at third line and beyond. Although these data were not available in the publications, they are crucial to fully understand the real-world efficacy and tolerability of treatment regimens and should be capture in future research. Further research is required in order to fully understand the treatment burden of SCLC therapies in real world, and to understand which treatment modes or regimens are associated with lower burden.

Although the development of new SCLC therapies is relatively limited, newly approved treatments are being added to treatment pathways. The National Comprehensive Cancer Network added nivolumab- and atezolizumab-based regimens to their treatment guidelines for SCLC.[74, 75] Despite this, novel therapies were not reflected in the real-world clinical environment captured as part of this SLR. This is likely due to the data collection methods used, e.g. while several years of data were examined in the publications, the data came from registries or patient records that pre-dated the introduction of immuno-therapies. Therefore, further research is required in order to fully understand the effectiveness and tolerability of novel therapies on SCLC in the real world, and to understand which treatment modes or regimens are associated with lower burden.

### 4.1 Limitations

Despite the applied SLR methodology, certain limitations were noted. While online registration of SLRs helps to increase data and methodological transparency and avoids the duplication of studies, this SLR was not registered online.[76] Despite this, a robust protocol has been created ahead of the SLR conduct and the research team has fully complied with the pre-specified research plan to avoid bias. Additionally, regarding the included data, the review itself was broad and covered a range of sources (‘real-world studies’) with varied characteristics, the captured data were fragmented, and only simple qualitative analysis of numerical data points could be conducted. A robust meta-analysis of data was not possible, because of known (and likely unknown) confounding factors between source publications.

As captured publications provided real-world data, in most cases the sampled patients had been allocated to treatment according to ‘physician opinion’. In some cases, after allocation, treatment changes or discontinuations occurred, again by physician or patient decision. Therefore, in many cases treatment allocation (or alteration) was open to bias, meaning outcomes data could not easily be compared.

A substantial proportion of the captured publications reported retrospective chart reviews or registry studies, meaning the examined data were inherently limited to those that were coded and entered at the time of treatment. Although details of all concomitant therapies or comorbidities would be useful for analysis, these data were not commonly available. Even in cases where such supplemental data were captured, these were not always reported: for example, although concomitant radiotherapy was often given alongside chemotherapy, the proportion of patients who actually received it was generally not described, and separate outcomes were generally not given for these patients.

Additionally, in cases where the line of therapy was not described, inferring the line of the given therapy may have introduced additional bias. However, without this step, data would have been further fragmented and more difficult to analyze.

The ROBINS-I tool for the assessment for bias in non-randomized studies was used in this SLR to examine the risk of bias in the captured observational studies. While no specific tools exist for the evaluation of bias in real-world studies, ROBINS-I was chosen based on its robust methodology (developed based on a Cochrane assessment tool for randomized studies), and its applicability to studies in which individuals who have received different interventions are followed up over time. ROBINS-I presented several limitations: the tool was developed based on the Cochrane questionnaire for assessing bias in randomized trials and as such, it closely follows this tool in assessing the specific domains of bias relevant to randomized studies. Therefore, not all parts of ROBINS-I were useful for evaluating real-world studies and the overall risk of bias calculated may not capture all the bias present in the studies. Additionally, the overall risk of bias was based on individual interpretation and judgement by the reviewer using the tool, therefore discrepancies could exist between reviewer opinions–this was corrected for by two reviewers assessing the bias separately. Lastly, the overall risk of bias was based on how comparable the non-randomized study was to a well-performed RCTs, therefore the overall results may be skewed to a high overall bias, e.g. as observational studies would not be expected to be as robust as RCTs, a low risk of bias was unlikely in these types of studies.

### 4.2 Conclusion

The publications included within this review assessed the effectiveness and tolerability of SCLC treatments in real-world setting. Generally, the extracted data were fragmented and inconsistently reported, providing only a limited potential for comparison owing to fundamental differences between studies. Therefore, more comparative research is needed to address this knowledge gap.

In addition, the captured publications were characterized by a moderate or serious risk of bias in several key aspects of the methodologies–e.g. missing data, confounding factors, incomplete reporting and unclear outcome measurements. However, as non-interventional studies are inherently less robust than RCT studies, it was unsurprising that many of the captured publications exhibited a moderate or high risk of bias. Despite this, the majority of the publications included statistical analyses to improve the robustness of conclusions. Future real-world observational studies should aim to minimize biases such as missing data or control for confounding factors (or fully address or adjust for this potential bias when reporting) and aim to collect robust data sets with full descriptions of the received treatments. Although there is an inherent variance in the methodology of real-world studies, caused by the data availability, the design of registry studies requires a more standardized and robust approach.

The available real-world evidence shows a limited number of treatment options in SCLC, all associated with short OS and TRAEs that increase the burden of the disease. These outcomes appeared poorer than those from RCTs, highlighting a real need for improved treatment options in SCLC.

Novel therapies, such as immune-checkpoint inhibitors, are being introduced into treatment guidelines and may offer a new direction in SCLC management.

## Supporting information

S1 Table OVID search strategies and numbers of results for SLR conducted 12th February 2018. HRQoL = health-related quality of life; SLR = systematic literature review.
(DOCX)

S2 Table OVID search strategies and numbers of results for SLR update conducted 19th November 2018. HRQoL = health-related quality of life; SLR = systematic literature review.
(DOCX)

S3 Table The detailed description of studies included in systematic literature review. ***Please note*:** *grey literature publications (non-peer-reviewed literature*, *e*.*g*. *abstracts*, *posters and conference proceedings) are presented in italics*. *Sources not in italics represents peer-reviewed full-text articles*. *No statistical analysis of demographics was presented. †No statistical analysis of results was presented. ‡Follow-up duration was not defined. CAV = cyclophosphamide, doxorubicin and vincristine; CEI = cisplatin, etoposide and ifosfamide; CM = cranial metastasis; CNS = central nervous systems; CR = complete response; CTCAE = Common Terminology Criteria for Adverse Events; ECOG = Eastern Cooperative Oncology Group; ED = extensive disease; ERCC1 = excision repair cross-complementation group 1; G-CSF = granulocyte-colony stimulating factor; HR = hazard ratio; HUS = health utility score; ILD = interstitial lung disease; Ipi = ipilimumab; LD = limited disease; Nivo = nivolumab; NR = not reported; ORR = objective response rate; OS = overall survival; PCI = prophylactic cranial irradiation; PFS = progression-free survival; PR = partial response; RR = response rate; SCLC = small-cell lung cancer; SEER = Surveillance, Epidemiology and End Results; TBC = to be confirmed; TRAEs = treatment-related adverse events; TRT = thoracic radiotherapy; TTP = time to progression; UK = United Kingdom; US = United States.
(DOCX)

S4 Table List of captured publications for which lines of therapy were inferred, and the rationale in each case. *‘Urgent chemotherapy’ was defined as chemotherapy administered in an intensive-care unit to treat organ failure related to cancer. **Grey literature was defined as non-peer-reviewed literature, e.g. abstracts, posters and conference proceedings. CM = cranial metastasis; ED = extensive disease; LD = limited disease; OS = overall survival; PCI = prophylactic cranial irradiation; SCLC = small-cell lung cancer.
(DOCX)

S5 Table PRISMA checklist. (DOC)

## Abbreviations

2L: second line
CAV: cyclophosphamide, doxorubicin and vincristine
CEI: cisplatin, etoposide and ifosfamide
CM: cranial metastasis
CNS: central nervous system
CR: complete response
CT: chemotherapy
CTCAE: Common Terminology Criteria for Adverse Events
ECOG: Eastern Cooperative Oncology Group
ED: extensive disease
ERCC1: excision repair cross-complementation group 1
ERCC1+: excision repair cross-complementation group 1 gene positive
FDA: Food and Drug Administration
G-CFS: granulocyte-colony stimulating factor
HR: hazard ratio
HRQoL: health-related quality of life
HUS: health utility score
ILD: interstitial lung disease
LD: limited disease
NA: not available
NR: not reported
ORR: objective response rate
OS: overall survival
PCI: prophylactic cranial irradiation
PD: progressive disease
PFS: progression-free survival
PR: partial response
PRISMA: Preferred Reporting Items for Systematic Reviews and Meta-Analyses
RCT: randomized controlled trial
ROBINS-I: Risk of Bias in Non-Randomized Studies of Interventions questionnaire
SCLC: small-cell lung cancer
SD: stable disease
SEER: Surveillance, Epidemiology and End Results
SLR: systematic literature review
TRAEs: treatment-related adverse events
TRT: thoracic radiotherapy
TTP: time to progression
UK: United Kingdom
US: United States
