# Supplementary material for: Real-world effectiveness and tolerability of small-cell lung cancer (SCLC) treatments: A systematic literature review (SLR)
Source: PLoS One. 2019 Jul 18;14(7):e0219622. doi: 10.1371/journal.pone.0219622 (PMC6638917; doi:10.1371/journal.pone.0219622)
Supplement: S2 Table — HRQoL = health-related quality of life; SLR = systematic literature review. (DOCX) [file pone.0219622.s002.docx]

**S2 Table. OVID search strategies and numbers of results for SLR update conducted 19^th^ November 2018**

| Search term | Results |
| --- | --- |
| 1. **Search for clinical endpoints** | |
| SCLC or small cell lung cancer or oat cell carcinoma or combined cell carcinoma or small cell carcinoma | 187,089 |
| **AND** | |
| Radiotherapy/ or antineoplastic agent/ or lung surgery/ or multimodality cancer therapy/ or chemotherapy/ or nivolumab/ or topotecan/ or amrubicin/ or ipilimumab/ or CAV/ or cyclophosphamide/ or rovalpituzumab tesirine/ or vinorelbine/ or paclitaxel/ or docetaxel/ or mitomycin/ or epirubicin/ or belotecan/ or bendamustine/ or ifosfamide/ or doxorubicin/ or vincristine/ or cisplatin/ or carboplatin/ or etoposide/ or irinotecan/ or gemcitabine/ or ziv-aflibercept/ or supportive care/ or palliative therapy or consolidation or disease management | 1,610,684 |
| **AND (one of the following)** | |
| Efficacy or effectiveness or tolerability or response or objective response rate or overall response rate or overall survival or partial response or partial remission or stable disease or progressive disease or overall survival or progression free survival or PFS or time or response or duration of response or improvement to disease related symptoms | 15,763,057 |
| (((Ae or safe or safety or side effect$ or adverse or undesirable or harm$ or serious or toxic) adj3 effect$) or reaction$ or event$ or outcome$).mp.  AND Adverse drug reaction/ or drug toxicity/ or drug safety / or drug monitoring/ or drug hypersensitivity/ or postmarketing surveillance/ or drug surveillance program/ or phase iv clinical trial/ or toxicity.mp. or complication$.mp. or noxious.mp. or tolerability.mp. or postoperative complication/ or Preoperative Complication/ | 7,474,082 |
| **AND** | |
| ((observational study.pt. or observational study as topic.mh. or case-control studies.mh. or cohort studies.mh. or cross-over studies.mh. or epidemiologic studies.mh. or cross-sectional studies.mh. or evaluation studies as topic.mh. or meta-analysis as topic.mh. or practice guidelines as topic.mh. or case control.tw. or case controlled.tw. or case controls.tw. or cohort.tw. or cohorts.tw. or follow-up.tw. or followup.tw. or longitudinal.tw. or matched-pair analysis.mh. or observational studies.tw. or observational study.tw. or multicenter study.pt.) and administrative data.tw.) or administrative database.tw. or administrative databases.tw. or chart review.tw. or data registry.tw. or data registries.tw. or databases, factual.mh. or medical record linkage.mh. or medical record review.tw. or medical records systems, computerized.mh. or national database.tw. or observational analysis.tw. or patient-reported outcomes measurement information system.tw. or promis.tw. or patient registry.tw. or patient registries.tw. or practice based research network.tw. or practice based research networks.tw. or pbrn.tw. or pbrns.tw. or registries.mh. or national hospital discharge survey.tw. or family study/ or real world.mp NOT randomized controlled trial .pt. | 291,817 |
| **Resulting in:** | |
| Initial total | 552 |
| Limits applied (English language, 2018–present) | 100 |
| **Total (de-duplicated)** | **97** |
|  |  |
| 1. **Search for HRQoL endpoints** | |
| SCLC or small cell lung cancer OR oat cell carcinoma OR combined cell carcinoma OR small cell carcinoma | 187,089 |
| **AND** | |
| ((observational study.pt. or observational study as topic.mh. or case-control studies.mh. or cohort studies.mh. or cross-over studies.mh. or epidemiologic studies.mh. or cross-sectional studies.mh. or evaluation studies as topic.mh. or meta-analysis as topic.mh. or practice guidelines as topic.mh. or case control.tw. or case controlled.tw. or case controls.tw. or cohort.tw. or cohorts.tw. or follow-up.tw. or followup.tw. or longitudinal.tw. or matched-pair analysis.mh. or observational studies.tw. or observational study.tw. or multicenter study.pt.) and administrative data.tw.) or administrative database.tw. or administrative databases.tw. or chart review.tw. or data registry.tw. or data registries.tw. or databases, factual.mh. or medical record linkage.mh. or medical record review.tw. or medical records systems, computerized.mh. or national database.tw. or observational analysis.tw. or patient-reported outcomes measurement information system.tw. or promis.tw. or patient registry.tw. or patient registries.tw. or practice based research network.tw. or practice based research networks.tw. or pbrn.tw. or pbrns.tw. or registries.mh. or national hospital discharge survey.tw. or family study/ or real world.mp NOT randomized controlled trial .pt. | 291,817 |
| **AND** | |
| Patient reported outcomes or PRO or PROs or EORTC QLC-C30 or EORTC QLQ-LC13 or LCSS or EQ-5D or HRQoL or Activities of daily living or Emotional Functioning or Cognitive functioning or Physical functioning or Social Functioning or preference based measure or health utility or time trade-off or standard gamble | 795,000 |
| **Resulting in:** | |
| Initial total | 79 |
| Limits applied (English language, 2018–present) and de-duplicated | 16 |
| **Total (de-duplicated)** | **15** |

HRQoL = health-related quality of life; SLR = systematic literature review.
