# Supplementary material for: Real-world effectiveness and tolerability of small-cell lung cancer (SCLC) treatments: A systematic literature review (SLR)
Source: PLoS One. 2019 Jul 18;14(7):e0219622. doi: 10.1371/journal.pone.0219622 (PMC6638917; doi:10.1371/journal.pone.0219622)
Supplement: S3 Table — Please note: grey literature publications (non-peer-reviewed literature, e.g. abstracts, posters and conference proceedings) are presented in italics. Sources not in italics represents peer-reviewed full-text articles. *No statistical analysis of demographics was presented. †No statistical analysis of results was presented. ‡Follow-up duration was not defined. CAV = cyclophosphamide, doxorubicin and vincristine; CEI = cisplatin, etoposide and ifosfamide; CM = cranial metastasis; CNS = central nervous systems; CR = complete response; CTCAE = Common Terminology Criteria for Adverse Events; ECOG = Eastern Cooperative Oncology Group; ED = extensive disease; ERCC1 = excision repair cross-complementation group 1; G-CSF = granulocyte-colony stimulating factor; HR = hazard ratio; HUS = health utility score; ILD = interstitial lung disease; Ipi = ipilimumab; LD = limited disease; Nivo = nivolumab; NR = not reported; ORR = objective response rate; OS = overall survival; PCI = prophylactic cranial irradiation; PFS = progression-free survival; PR = partial response; RR = response rate; SCLC = small-cell lung cancer; SEER = Surveillance, Epidemiology and End Results; TBC = to be confirmed; TRAEs = treatment-related adverse events; TRT = thoracic radiotherapy; TTP = time to progression; UK = United Kingdom; US = United States. (DOCX) [file pone.0219622.s003.docx]

**S3 Table. The detailed description of studies included in systematic literature review. *Please note:*** *grey literature publications (non-peer-reviewed literature, e.g. abstracts, posters and conference proceedings) are presented in italics. Sources not in italics represents peer-reviewed full-text articles.*

| Author | Methodology/setting/follow-up duration (if available) | Population | n | Line of therapy | Therapy | Outcomes | Conclusion |
| --- | --- | --- | --- | --- | --- | --- | --- |
| *Al Farsi et al. 2017*^†‡^[38]* | - *A retrospective chart review of SCLC patients diagnosed between 1^st^ January 2011 and 31^st^ December 2014 in Canada* - *This study aimed to explore patterns of relapse, and utility of PCI* | *SCLC;*  *aged 66 (median)* | *229* | *First line* | *Chemotherapy and PCI* | - *RR (PR and CR) for overall population* - *Median OS for overall population, ED- and LD-SCLC* - *Median PFS for overall population, ED- and LD-SCLC* - *Median time to CNS relapse for overall population, patients who receive PCI and patients who did not* | - *CNS relapse occurs frequently and more commonly in patients who do not receive PCI* - *Implementation of PCI in routine clinical practice appears to influence patterns of recurrence* |
| *Aquin et al. 2018^‡^[51]* | - *This retrospective chart review used the population-based Manitoba Cancer Registry to identify SCLC patients diagnosed between 2004 and 2013 in Manitoba and treated with chemotherapy* - *This study aimed to explore the survival outcomes in patients treated with carboplatin and cisplatin* - *Statistical analysis of demographics was conducted but the type of test was not defined* - *Statistical analysis of results: Cox proportional hazard methods, logistic regression* | *SCLC; age not reported* | *531* | *First line* | *Carboplatin and cisplatin* | - *Median OS for patients treated with cisplatin versus carboplatin* - *TRAEs for patients treated with cisplatin versus carboplatin* | - *Carboplatin appears to be an equally effective treatment option for SCLC, facilitating equivalent survival while avoiding toxicity* |
| Behera et al. 2016^‡^[28] | - Retrospective database analysis identified patients through SEER and Medicare databases diagnosed in the US between 1985 and 2005 - This study aimed to evaluate trends in real-world efficacy of available systemic therapies and the impact on outcomes in the US - Statistical analysis of demographics: Wilcoxon rank-sum test for age and with the χ^2^ test or Fisher’s exact test for the categorical covariates as appropriate - Statistical analysis of results: logistic regression modelling, Cox proportional hazard modelling, and log-rank testing of Kaplan-Meier results | LD- and ED-SCLC;  aged 71 (median) | 47,351 | First line | Chemotherapy | - Median OS for patients treated with chemotherapy versus patients who did not receive chemotherapy - Median OS for patients treated with cisplatin regimens or carboplatin regimens - Median OS for patients treated with platinum-contain regimens or other regimens | - There is a clinical benefit from chemotherapy (platinum-containing and salvage) in SCLC patients treated in the real world |
| Boni et al. 2015*[30] | - This retrospective single-institution study included consecutive patients presenting to the Medical Oncology Unit of Reggio Emilia (Italy) between December 1998 and December 2008 treated at first line with CEI regimen - Patients with CNS metastases were included in this study - This study aimed to evaluate the activity of CEI regimen in SCLC patients - Statistical analysis of results: log-rank testing of Kaplan-Meier results - Follow-up duration: 5 years | SCLC;  aged 63 (median) | 46 | First line | CEI regimen with possible radiotherapy | - Median OS for overall population, LD and ED - ORR for overall population, LD and ED - Mortality for overall population, LD and ED - TTP for overall population, LD and ED - TRAEs for overall population | - CEI regimen gave interesting median OS results both, LD- (28.2 months) and ED-SCLC (11.8 months) - In both stages, the OS and TTP appear higher than expected, comparing to the results obtained with cisplatin-etoposide regimen published in the literature - In the absence of a firm evidence of any activity of biological agents, CEI regimen may be a possible therapeutic option, with high activity and an acceptable toxicity profile |
| *Cope et al. 2018^‡^[60]* | - *This retrospective registry study evaluated data from Flatiron Health database from January 2011 to September 2017 for patients with SCLC treated in third line* - *This study aimed to estimate the comparative efficacy of nivo ± ipi versus current standard of care for SCLC patients treated in third line in the US* - *The ‘real-world’ cohort was constructed with demographic criteria matching those of CheckMate 032* - *Statistical analysis of results: fractional polynomial models, and a Cox regression model weighted by use of a logistic propensity score model* | *SCLC; aged 64 (mean) or 64 (median)* | *92* | *Third line* | *Chemotherapy (predominantly topotecan) versus nivo ± ipi (results as reported in CheckMate 032 trial)* | - *Median OS for third-line patients treated with chemotherapy versus patients treated with nivo ± ipi in CheckMate 032 trial* - *Mortality for third-line patients treated with chemotherapy versus patients treated with nivo ± ipi in CheckMate 032 trial* | - *These analyses suggest that nivo ± ipi is more efficacious than standard of care for third-line patients with SCLC in terms of OS* |
| de Jong et al. 2006^†‡^[52] | - This retrospective chart review included consecutive patients with SCLC diagnosed between January 1996 and July 2004 at the University Medical Center Groningen - The aim of this study was to analyze the efficacy of chemotherapy used in third line patients with recurrent SCLC - Statistical analysis of demographics: Mann-Whitney U-tests | SCLC; aged 58 (median) | 191 | First line | Chemotherapy with possible radiotherapy | - ORR in patients treated with chemotherapy | - One out of six patients received third-line chemotherapy for recurrent SCLC and it was effective in one out of four patients - It was suggested that this response warrants the use of third-line chemotherapy in selected patients - Future studies of  cost-effectiveness analysis are needed |
|  |  |  | 94 | Second line |  | - ORR in patients treated with chemotherapy |  |
|  |  |  | 35 | Third line | - Cyclophosphamide/ doxorubicin/ etoposide - Carboplatin and paclitaxel, with or without ifosfamide - Cisplatin combinations - Gemcitabine monotherapy - Gemcitabine/ carboplatin - Teniposide - Other | - Median OS in third-line chemotherapy patients - Mortality in third-line chemotherapy patients - ORR in patients treated with chemotherapy |  |
| Fisher et al. 2012[34] | - Retrospective population-based chart review of Alberta Cancer Registry identified patients diagnosed in years 2004 and 2008 in Canada - This study aimed to describe the receipt of chemotherapy provided to elderly SCLC patients in Alberta and asses the tolerance and survival - Statistical analysis for associations between demographics and treatment: χ^2^ test or Fisher’s exact test (if the expected value of a cell was less than five) - Statistical analysis of results: Cox proportional hazard modelling with χ^2^ testing, and log-rank and Wilcoxon testing of Kaplan-Meier results - Follow-up duration: up to 6 years (patients were followed until either death or 31^st^ December 2010) | LD- and ED-SCLC,  aged ≥75 | 117 | First line | Chemotherapy | - Median OS for patients who completed all treatment cycles versus patients who did not - Adjusted HR for death for elderly versus very elderly patients | - Elderly patients (≥80), compared to those 75–79 years of age, received less chemotherapy and were more likely to receive a dose reduction, but were equally likely to complete all chemotherapy cycles - Significant proportion of elderly patients were able to withstand chemotherapy and receive a survival benefit even with dose reduction - Elderly patients should be considered for treatment with chemotherapy |
| Garassino et al. 2011*^‡^[62] | - This retrospective chart review included cases of patients with SCLC treated with first-line platinum chemotherapy receiving second-line chemotherapy. These cases were submitted by the investigators - This study aimed to explore the clinical outcomes of second-line therapy - Statistical analysis of results: Cox proportional hazard modelling | SCLC; aged 63 (median) | 161 | First line | Platinum-etoposide chemotherapy | - RR for patients that responded to first-line platinum-based chemotherapy | - This study suggests that second-line chemotherapy should be selected based on ECOG-Performance status and the response to first line |
|  |  |  |  | Second line | Chemotherapy | - Median OS for patients receiving second-line therapy - Median PFS for patients receiving second-line therapy - ORR for patients receiving second-line therapy |  |
| Genestreti et al. 2015*^‡^[63] | - This retrospective chart review included data of all consecutive patients with SCLC treated in 7 institutions (4 in Italy, 1 in the UK, 1 in Turkey and 1 in Japan) between January 2007 and December 2011 - This study aimed to evaluate the clinical outcomes in platinum-sensitive patients with relapsed SCLC treated with platinum/etoposide re-challenge - Statistical analysis of results: Cox proportional hazard modelling, and log-rank testing of Kaplan-Meier results | SCLC; platinum sensitive; aged 64 (median) | 112 | First line | Platinum/etoposide | - Relapse-free survival for first-line patients - ORR for first-line patients | - This study confirms interesting results in platinum-sensitive patients treated with re-challenge of first-line chemotherapy |
|  |  |  | 112 | Second line | Platinum/etoposide re-challenge | - Median OS from diagnosis for patients re-challenged with platinum/etoposide in second line - Median OS from re-challenge for patients re-challenged with platinum/etoposide in second line - Median PFS for patients re-challenged with platinum/etoposide in second line - ORR for second-line patients - Mortality from the start of re-challenge therapy |  |
|  |  |  | 40 | Third and further lines | - CAV - Topotecan - Other - Carboplatin/ etoposide - Cyclophosphamide/ epirubicin/ vincristine - Cisplatin/etoposide - Carboplatin/ irinotecan - Gemcitabine/ paclitaxel | - Median OS for patients treated with anthracycline-based regimen in third line - Median OS for patients treated with platinum/etoposide in third line - Median OS for patients treated with topotecan in third line - Median PFS for patients treated with anthracycline-based regimen in third line - Median PFS for patients treated with platinum/etoposide in third line - Median PFS for patients treated with topotecan in third line |  |
| *Hennemann*  *et al. 2009*^†‡^[44]* | - *This retrospective review examined cancer patients in Brazil* - *This study aimed to evaluate the correlation between ERCC1 expression and OS* | *LD- and ED-SCLC;*  *aged 60.8 (median)* | *42* | *First line* | *Cisplatin-based regimen and possible concomitant radiotherapy* | - *Median OS for LD- and ED-SCLC* | - *ERCC1 expression does not correlate with survival* |
| Imai et al. 2017*^‡^[36] | - Retrospective cohort study identified patients treated with platinum-based regimen at the first line and amrubicin at second was at four Japanese institutions between November 2003 and September 2015 - The aim of this study was to assess the feasibility of amrubicin monotherapy in relapsed elderly patients - Statistical analysis of results: unspecified univariate and multivariate methods, including log-rank testing | SCLC;  aged ≥70 | 86 | Second line | Amrubicin | - Median PFS for overall population - Median OS for overall population - TRAEs for overall population - Median PFS for sensitive versus relapsed patients - Median OS for sensitive versus relapsed patients | - Amrubicin monotherapy may be an effective and tolerable regimen for elderly patients with previously treated SCLC |
| *Itotani et al. 2017*^†‡^[47]* | - *Retrospective chart review performed at the Tazuke Kofukai Medical Research Institute Kitano Hospital (Japan) between January 2011 and December 2016* - *This study aimed to investigate the efficacy and safety of carboplatin and weekly paclitaxel regimen* | *SCLC; patients with ILD; aged 70 (mean)* | *21* | *Mixed line* | *Carboplatin and weekly paclitaxel* | - *RR and disease control rate for overall population* - *Median PFS for overall population* - *Median OS for overall population* - *TRAEs for overall population* | - *Carboplatin and weekly paclitaxel regimen was effective in SCLC patients with ILD who were previously treated with more than one cytotoxic chemotherapy regimen; however, 19% patients experienced exacerbation of ILD* |
| Labbe et al. 2017^‡^[29] | - Longitudinal cohort study evaluated out-patients across various disease states - Participants completed a demographic survey, EQ-5D-3L and Patient-reported Outcomes -CTCAE - The aim was to compare HUS by mutational status, therapy, response to treatment, and severity of symptoms - Statistical analysis of demographics: bivariate analyses across groups using the χ^2^ and non-parametric Mann-Whitney U tests - Statistical analyses of results: Wilcoxon and Mann-Whitney U testing (*for HUS*), Kruskal-Wallis and Mann-Whitney U testing (*for TRAEs*), and Spearman correlation (*between HUS and TRAEs*) | SCLC;  aged 66 (median) | 30 | First line | Chemotherapy | - TRAEs for the overall population - HUS for the overall population | - SCLC patient group was too small for make any meaningful conclusions regarding the relationship between TRAEs and HUS |
| *Lattuca-Truc*  *et al. 2018*^†‡^[54]* | - *This retrospective registry study included consecutive patients with SCLC identified through a database from the weekly multidisciplinary thoracic oncology board of Grenoble University Hospital revisited from 1997 to 2017* - *This study aimed to assess whether the characteristics of patients with SCLC and their responses to chemotherapy changed over time* | *SCLC; aged 64 years (median); range: 56-71* | *499* | *First line* | *Etoposide/platinum* | - *Median OS from the onset of treatment* - *RR in metastatic patients* | - *This study suggests that there was no improvement in survival or RR to chemotherapy in patients with SCLC since 1997* |
|  |  |  | *NR* | *Second line* | *Etoposide/platinum or taxane* | - *RR in all patients* |  |
|  |  |  | *NR* | *Third line* | *Regimen not specified* | - *RR in all patients* |  |
| *Maenpaa et al. 2010*^†‡^[42]* | - *Prospective observational study conducted in Europe, Australia and Canada enrolled cancer patients between December 2007 and October 2009* - *The aim of this study was to evaluate real-world G-CSF prophylaxis and neutropenia-related outcomes in patients receiving chemotherapy for solid tumors who were assessed as high risk for febrile neutropenia* | *SCLC;*  *aged 62 (median)* | *1.370* | *First line* | *Chemotherapy (mainly platinum/etoposide)* | - *TRAEs for ED-SCLC* | - *Febrile neutropenia incidence was highest in the SCLC patients, compared to other group of patients* |
| Noguchi et al. 2010[35] | - Retrospective chart review identified patients admitted to Tokyo Metropolitan Geriatric Hospital (Division of Respiratory Medicine; Japan) between January 1996 and March 2007 using the database of pathologic records - This study aimed to assess the efficacy and safety of anticancer treatment for very elderly patients. - Statistical analysis of demographics was conducted but the type of test was not defined - Statistical analysis of results: log-rank testing of Kaplan-Meier results - Follow-up duration: ≥2 years | LD- and ED-SCLC;  aged 70–79 and ≥80 | 83 | First line | Chemotherapy and radiotherapy | - Median OS for treated and untreated patients (both age groups) - Median OS for LD, ED, elderly, very elderly - ORR for LD, ED, elderly, very elderly - 1-year survival rate for LD, ED, elderly, very elderly - 2-year survival rate for LD, ED, elderly, very elderly - TRAEs for elderly versus very elderly | - There were no significant differences in ORR, survival rate, or toxicities between patients aged 80 years and those aged 70–79 years - Carboplatin-based combination chemotherapies with or without TRT can be considered for the initial treatment of patients aged ≥80 |
| *Schellenberg*  *et al. 2010*^‡^[43]* | - *This retrospective chart review obtained data for patients diagnosed between January 2006 and December 2008 in British Columbia who were eligible for PCI* - *This study aimed to determine the proportion of patients developing CM following PCI, the median time from diagnosis to CM and the median survival post CM* - *Statistical analysis of results was conducted but the type of test was not defined* | *SCLC;*  *age NR* | *28* | *Second line* | *Brain re-irradiation, chemotherapy, surgery, steroids* | - *Median time from CM to death for overall population* - *Median OS from CM for re-irradiation versus surgery or chemotherapy versus steroids* | - *Post PCI re-irradiation is safe in patients with CM* - *Re-irradiation may provide survival benefits compared to other forms of treatment* |
| *Schwartzberg et al. 2018^†‡^[68]* | - *Retrospective analysis of Flatiron Health database between 1^st^ January 2011 and 30^th^ September 2017 in the US* - *The aim of this study was to evaluate  second-line patterns of care and outcomes attempts to contextualize CheckMate 032 data by creating a matched real-world comparator* - *The ‘real-world’ cohort was constructed with demographic criteria matching those of CheckMate 032* | *SCLC;*  *aged 65 (mean)* | *628* | *Second line* | *Systemic treatment (full cohort)* | - *Median OS for overall population,  platinum-sensitive and  platinum-resistant patients* - *One-year survival for overall population, platinum-sensitive and platinum-resistant patients* - *Two-year survival for overall population, platinum-sensitive and platinum-resistant patients* | - *This large retrospective study demonstrates poor survival in second-line patients* - *Results from CheckMate 032 present immuno-oncology therapy as a potential option for patients with SCLC* |
|  |  |  | *254* | *Second line* | *Systemic treatment (matched cohort)* | - *Median OS for overall population, platinum-sensitive and platinum-resistant patients* - *One-year survival for overall population, platinum-sensitive and platinum-resistant patients* - *Two-year survival for overall population, platinum-sensitive and platinum-resistant patients* |  |
|  |  |  | *98* | *Second line* | *Nivo (3 mg/kg)* | - *Median OS for CheckMate 032 population* - *One-year survival for CheckMate 032 population* |  |
|  |  |  | *61* | *Second line* | *Nivo + Ipi (1 mg/kg + 3 mg/kg)* | - *Median OS for CheckMate 032 population* - *One-year survival for CheckMate 032 population* |  |
|  |  |  | *54* | *Second line* | *Nivo + Ipi (3 mg/kg + 1 mg/kg)* | - *Median OS for CheckMate 032 population* - *One-year survival for CheckMate 032 population* |  |
| *Schwartzberg et al. 2018^†‡^[49]* | - *Retrospective analysis of Flatiron Health electronic health records between 1^st^ January 2011 and 30^th^ September 2017 in the US* - *The aim of this study was to describe third-line outcomes in US patients with SCLC, and create a matched real-world historical comparator to CheckMate 032* - *Within the results, a ‘real-world’ cohort was constructed with demographic criteria matching those of CheckMate 032* | *SCLC;*  *aged 65 (mean)* | *218* | *Third line* | *Unspecified type of treatment (all third-line patients)* | - *Median OS* - *One-year OS rate* - *Eighteen-month OS rate* - *Two-year OS rate* | - *Poor survival among third-line patients with SCLC highlights the need for more effective and tolerable therapies* - *Nivo demonstrated improved outcomes in heavily pretreated patients when compared to real-world data* - *Nivo may represent a new therapeutic for patients with recurrent SCLC* |
|  |  |  | *92* | *Third line* | *Unspecified type of treatment (matched third-line cohort)* | - *Median OS* - *One-year OS rate* - *Eighteen-month OS rate* - *Two-year OS rate* |  |
|  |  |  | *TBC* | *Third line* | *To be presented after the conference* | - *CheckMate 032 data to be presented after the conference* |  |
| Simos et al. 2014*^‡^[15] | - This retrospective chart review included patients with SCLC who received at least 3 lines of chemotherapy between 1^st^ January 2000 and 31 December 2010 in five cancer centers (3 in Canada, 1 in the UK and 1 in Australia) - This study aimed to compare the outcomes of patients treated with third-line treatment to patients who received the best supportive care - Statistical analysis of results: Cox proportional hazard modelling, logistic regression modelling, and log-rank testing of Kaplan-Meier results | LD- and ED-SCLC; aged 61 (median) | 120 | First line | - Platinum-based regimens - Other | - Median PFS for all patients - ORR for all patients | - This study showed that only few patients receive three lines of chemotherapy - RR and OS are limited at third line and the clinical relevance of third-line therapy is not clear - Patients who responded to third line chemotherapy showed a significantly longer OS than patients who did not respond |
|  |  |  |  | Second line | - Platinum-based regimens - CAV-based regimens - Topotecan-based regimens - Other | - Median PFS for all patients - ORR for all patients |  |
|  |  |  |  | Third line | - Platinum-based regimens - CAV-based regimens - Topotecan-based regimens - Other | - Median OS for patients who responded at third line - Median OS for patients who did not respond at third line - Median OS for patients with LD-SCLC at diagnosis - Median OS for patients with ED-SCLC at diagnosis - Median OS for patients with partial or complete response at first line - Median OS for patients with stable or progressed disease at second line - Median PFS for all patients - Median PFS for patients who responded to third line - Median PFS for patients who did not respond to third line - Median PFS at third line for patients who went on to receive subsequent lines of therapy - ORR for all patients - ORR at third line for patients who went on to receive subsequent lines of therapy - Mortality for overall population |  |
| *von Eiff et al. 2018*^‡^[57]* | - *This retrospective chart review included patients with SCLC treated with paclitaxel between 2005 and 2015 at Thoraxklinik at Heidelberg University Hospital* - *This study aimed to evaluate prognostic factors and outcomes of patients with SCLC treated with paclitaxel* - *Statistical analyses of results: univariate and multivariate methods, including Kaplan-Meier analysis and multivariate Cox regression analysis* | *SCLC; aged 64 (median)* | *185* | *Mixed lines* | *Paclitaxel* | - *Median OS* - *Median PFS* - *ORR* - *Duration of response* - *TRAEs* | - *This study showed that paclitaxel was effective even in heavily pretreated patients* - *Patients should be selected for the treatment based on age, performance status, and metastases as paclitaxel can be particularly beneficial for patients in good condition* |
| Wheatley-Price et al. 2014*^‡^[37] | - A retrospective  single-institution chart review of patients hospitalized within the Ottawa Hospital Cancer Center (Canada) identified patients receiving palliative chemotherapy between April 2008 and January 2010 - The aim of this study was to assess the OS after palliative chemotherapy, place of discharge from hospital, and further chemotherapy received - Statistical analyses of results: Cox proportional hazard methods, and univariate and multivariate logistic regression | SCLC;  aged 61 (median) | 44 | First line | Palliative chemotherapy | - Median OS for SCLC population - Mortality rates at 30 days for SCLC - Mortality rates at discharged for SCLC | - Although the median survival was short, many patients remained well enough to be discharged or receive further treatment |

*No statistical analysis of demographics was presented. †No statistical analysis of results was presented. ‡Follow-up duration was not defined.

CAV = cyclophosphamide, doxorubicin and vincristine; CEI = cisplatin, etoposide and ifosfamide; CM = cranial metastasis; CNS = central nervous systems; CR = complete response; CTCAE = Common Terminology Criteria for Adverse Events; ECOG = Eastern Cooperative Oncology Group; ED = extensive disease; ERCC1 = excision repair cross-complementation group 1; G-CSF = granulocyte-colony stimulating factor; HR = hazard ratio; HUS = health utility score; ILD = interstitial lung disease; Ipi = ipilimumab; LD = limited disease; Nivo = nivolumab; NR = not reported; ORR = objective response rate; OS = overall survival; PCI = prophylactic cranial irradiation; PFS = progression-free survival; PR = partial response; RR = response rate; SCLC = small-cell lung cancer; SEER = Surveillance, Epidemiology and End Results; TBC = to be confirmed; TRAEs = treatment-related adverse events; TRT = thoracic radiotherapy; TTP = time to progression; UK = United Kingdom; US = United States.
