# Supplementary material for: Real-world effectiveness and tolerability of small-cell lung cancer (SCLC) treatments: A systematic literature review (SLR)
Source: PLoS One. 2019 Jul 18;14(7):e0219622. doi: 10.1371/journal.pone.0219622 (PMC6638917; doi:10.1371/journal.pone.0219622)
Supplement: S4 Table — *‘Urgent chemotherapy’ was defined as chemotherapy administered in an intensive-care unit to treat organ failure related to cancer. **Grey literature was defined as non-peer-reviewed literature, e.g. abstracts, posters and conference proceedings. CM = cranial metastasis; ED = extensive disease; LD = limited disease; OS = overall survival; PCI = prophylactic cranial irradiation; SCLC = small-cell lung cancer. (DOCX) [file pone.0219622.s004.docx]

S4 Table. List of captured publications for which lines of therapy were inferred, and the rationale in each case

| Author | Therapy | Inferred line | Rationale |
| --- | --- | --- | --- |
| **Peer-reviewed literature** | | | |
| Zerbib et al. 2017[27] | Urgent chemotherapy* | First | - The largest proportion of patients were newly diagnosed with pre-metastatic cancer |
| Behera et al. 2016[28] | Chemotherapy | First | - The largest proportion of patients had stage IV SCLC, with a median age of 71, and no previous surgery; therefore, it was inferred that the largest proportion of patients were newly diagnosed |
| Labbe et al. 2016[29] | Chemotherapy | First | - The largest proportion of patients had stage IV SCLC, with no treatment or receiving  first-line palliative systemic therapy; therefore, it was inferred that patients were receiving first-line therapy |
| **Grey literature**** | | | |
| Al Farsi et al. 2017[38] | Chemotherapy, radiotherapy | First | - The primary objective of the study was to determine patterns of first relapse. |
| Maenpaa et al. 2010[42] | Chemotherapy | First | - The largest proportion of patients had not received previous chemotherapy/radiotherapy; therefore, it was inferred that patients were receiving first-line therapy |
| Hennemann et al. 2009[44] | Chemotherapy with possible radiotherapy | First | - The largest proportion of patients had ED-SCLC, with a median age of 60.8, receiving chemotherapy/radiotherapy; therefore, it was inferred that patients were receiving first-line therapy |
| Schellenberg et al. 2010[43] | Radiation, surgery, chemotherapy, steroids | Second | - All patients received PCI at first line, and some patients received second-line therapy for CM; therefore, it was inferred that both first-line and second-line data were presented |
| Itotani et al. 2017[47] | Chemotherapy | Mixed | - Patients had previously received between two and six lines of therapy; therefore, it was inferred that patients were receiving a mixture of third-line therapy and beyond |

*‘Urgent chemotherapy’ was defined as chemotherapy administered in an intensive-care unit to treat organ failure related to cancer. **Grey literature was defined as non-peer-reviewed literature, e.g. abstracts, posters and conference proceedings.

CM = cranial metastasis; ED = extensive disease; LD = limited disease; OS = overall survival; PCI = prophylactic cranial irradiation; SCLC = small-cell lung cancer.
